# Radotinib Induces Apoptosis of CD11b^+^ Cells Differentiated from Acute Myeloid Leukemia Cells

**DOI:** 10.1371/journal.pone.0129853

**Published:** 2015-06-12

**Authors:** Sook-Kyoung Heo, Eui-Kyu Noh, Dong-Joon Yoon, Jae-Cheol Jo, Yunsuk Choi, SuJin Koh, Jin Ho Baek, Jae-Hoo Park, Young Joo Min, Hawk Kim

**Affiliations:** 1 Biomedical Research Center, Ulsan University Hospital, University of Ulsan College of Medicine, Ulsan, 682-060, Republic of Korea; 2 Department of Hematology and Oncology, Ulsan University Hospital, University of Ulsan College of Medicine, Ulsan, 682-714, Republic of Korea; 3 Department of Hematology and Oncology, Myongji Hospital, Gyeonggi-do, 412-270, Republic of Korea; University of Kansas Medical Center, UNITED STATES

## Abstract

Radotinib, developed as a BCR/ABL tyrosine kinase inhibitor (TKI), is approved for the second-line treatment of chronic myeloid leukemia (CML) in South Korea. However, therapeutic effects of radotinib in acute myeloid leukemia (AML) are unknown. In the present study, we demonstrate that radotinib significantly decreases the viability of AML cells in a dose-dependent manner. Kasumi-1 cells were more sensitive to radotinib than NB4, HL60, or THP-1 cell lines. Furthermore, radotinib induced CD11b expression in NB4, THP-1, and Kasumi-1 cells either in presence or absence of all trans-retinoic acid (ATRA). We found that radotinib promoted differentiation and induced CD11b expression in AML cells by downregulating LYN. However, CD11b expression induced by ATRA in HL60 cells was decreased by radotinib through upregulation of LYN. Furthermore, radotinib mainly induced apoptosis of CD11b^+^ cells in the total population of AML cells. Radotinib also increased apoptosis of CD11b^+^ HL60 cells when they were differentiated by ATRA/dasatinib treatment. We show that radotinib induced apoptosis via caspase-3 activation and the loss of mitochondrial membrane potential (*ΔΨ_m_*) in CD11b^+^ cells differentiated from AML cells. Our results suggest that radotinib may be used as a candidate drug in AML or a chemosensitizer for treatment of AML by other therapeutics.

## Introduction

Radotinib (Supect; C_27_H_21_F_3_N_8_OHCl; IY5511HCl; 4-Methyl-N-[3-(4-methyl-imidazol-1-yl)-5-trifluoromethyl-phenyl]-3-(4-pyrazin-2-yl-pyrimidin-2-ylamino)-benzamide hydrochloride; PubChem CID 16063245) is a BCR-ABL1 tyrosine kinase inhibitor (TKI). A phase II clinical trial evaluated the safety and efficacy of radotinib for patients with chronic phase chronic myeloid leukemia (CP-CML) with resistance and/or intolerance to other BCR-ABL1 TKIs and concluded that radotinib was well tolerated and effective. Radotinib is approved as a second-line treatment of CP-CML in South Korea [1]. A phase III study comparing radotinib with imatinib in patients newly diagnosed with TKI-naïve chronic phase CML (CP-CML) is now ongoing.

Acute myeloid leukemia (AML) is a cancer of the myeloid lineage of blood cells. Generally, it is characterized by the rapid growth of abnormal white blood cells that accumulate in the bone marrow and/or peripheral blood. AML is the most common type of acute leukemia in adults and its incidence increases with age. It is considered as a diverse malignancy defined by defective differentiation and accumulation of proliferative blasts [2]. Its treatment remains challenging, therefore development of more effective chemotherapeutic agents is urgently required [3].

Recently, there have been several reports about the ability of epidermal growth factor receptor inhibitors (EGFRIs) and BCR-ABL1 TKIs to induce differentiation or cell death of AML cells, which is generally considered as an off-target effect. For example, the EGFRI gefitinib induced myeloid differentiation of AML cell lines [4]. Another EGFRI, erlotinib, exhibited antineoplastic off-target effects in AML and myelodysplastic syndrome (MDS) [5]. Moreover, it killed CD34^+^ bone marrow blasts from patients with MDS and AML. Furthermore, gefitinib and erlotinib enhanced differentiation and cell cycle arrest induced by retinoic acid and vitamin D3 in AML cells [6]. In our previous studies, gefitinib enhanced differentiation induced by arsenic trioxide in the acute promyelocytic leukemia (APL) cell line [7]. Furthermore, the first generation BCR-ABL1 TKI for chronic myeloid leukemia (CML) imatinib (STI571, Gleevec) potentiated pharmacological activity of retinoic acid in APL cells and affected degradation of RARα and PML-RARα [8–10]. Dasatinib has been well-known as a multi-targeted kinase inhibitor against wild-type BCR-ABL and SRC family kinases [11,12]. It also promoted ATRA-induced differentiation of AML cells [2]. Furthermore, dasatinib enhanced p53-mediated targeting of human AML stem cells by chemotherapeutic agents [13]. Notably, dasatinib is considered to be a promising component of combinations with other chemotherapeutic agents against solid tumors, including breast, prostate, and other cancers [14]. In addition, dasatinib accelerated valproic acid-induced AML cell death by regulating differentiation capacity [12].

Although the mechanism of radotinib action mainly involves inhibition of the BCR-ABL1 tyrosine kinase and platelet-derived growth factor receptor (PDGFR), radotinib can exert many off-target effects on AML cells similar to many other TKIs [1]. However, the impact of radotinib on AML cells is largely unknown. We hypothesized that radotinib could induce differentiation or death of AML cells. The aim of this study, therefore, was to monitor the anti-leukemic effect of radotinib and to clarify mechanisms of its action on AML cells.

## Materials and Methods

### Reagents

RPMI 1640 medium and fetal bovine serum (FBS) were obtained from Gibco BRL (Grand Island, NY). The CellTiter 96 AQueous One Solution Cell Proliferation Assay (MTS) was purchased from Promega (Madison, WI). Anti-human CD11b-PE and Apoptosis Detection Kit I were purchased from BD Bioscience (San Diego, CA). Antibodies against ERK, phospho-SFK Y416, c-Raf, cleaved caspase-3, and anti-rabbit IgG-HRP were obtained from Cell Signaling Technology (Beverly, MA). Antibodies for western blot against LYN and β-actin were purchased from Santa Cruz Biotechnology (Santa Cruz, CA).

### High performance liquid chromatography (HPLC) analysis of radotinib

Twenty-five milligrams of radotinib were dissolved in 25 mL dimethylsulfoxide (DMSO). Then, a test solution containing 7.5 mL of the DMSO solution and 17.5 mL methanol was made. To prepare a reference standard solution, 4.5 mg radotinib was dissolved in 10 mL DMSO and 0.1 mL of the resulting solution was dissolved in 99.9 mL methanol. Liquid chromatography to measure peak area of radotinib was performed using 10-μL injection volumes of test and standard solutions as described below. To prepare the buffer solution, 1.2 mL acetic acid and 0.25 g of ammonium acetate were dissolved in water to a total volume of 1.0 L and pH was adjusted to 4.0 using HCl and sodium hydroxide. Liquid chromatography analysis was performed using a Hitachi L-2000 system (Hitachi High Technologies America, Inc., Pleasanton, CA). It was equipped with a UV 254 nm detector and a Shiseido Capcell Pak MGII column (C18, 5 μm, 4.6 × 150 mm). The solvent included [Ammonium acetate buffer (pH 4.0, A)/Methanol (B) 60:40 (0 min)], [A (60→ 20): B (40→ 80) (0–8 min)], [20: 80 (8–13 min)], [A (20→ 60): B (80→ 40) (13–13.1 min)], [60:40 (13.1–22 min)], flow rate 1.0 mL/min. Injection volume was 10 μL.

### Cell culture

Human AML HL60, THP-1, Kasumi-1, and NB4 cell lines were obtained from the American Type Culture Collection (ATCC, Manassas, VA). HL60, THP-1, and NB4 cells were grown as suspension cultures in 100-mm culture dishes in the RPMI-1640 medium supplemented with 10% heat-inactivated FBS and 1% penicillin-streptomycin in a 5% CO_2_ humidified atmosphere at 37°C. Kasumi-1 cells were also grown as suspension cultures in the RPMI-1640 medium, but were supplemented with 20% heat-inactivated FBS, 4.5 g/L glucose, 2 mM L-glutamine, and 1% penicillin-streptomycin.

### Patient samples

All patients were newly diagnosed AML or CML cases at Ulsan University Hospital, Ulsan, South Korea as described in Table 1. Blood or bone marrow samples were collected prior to their first round of chemotherapy.

**Table 1:** Information of patients.

UPN	Sex	Age	Disease	Cell source	Blast (%)	Karyotype
**1**	**F**	**80**	**AML without maturation**	**BM**	**72**	**46,XX,del(1)(p36.1),-7,add(9)(q12),-17,+2mar[13]/46,XX,add(9)(q22)[3]/46,XX[9]**
**2**	**M**	**27**	**AML with RUNX1/RUNX1T1**	**BM**	**25**	**46,XY,t(8;21)(q22;q22),inv(9)(p12q13)[16]/46,XY,inv(9)(p12q13)[4]**
**3**	**M**	**76**	**AML with maturation**	**BM**	**40**	**46,XY[20]**
**4**	**F**	**83**	**AML without maturation**	**BM**	**44**	**46,XX,del(5)(q21q35),-17,der(17;21)(q10;q10),+add(21)(q22),+mar[16]/ 46,idem,der(21;22)(q10;q10)[4]**
**5**	**F**	**44**	**AML, pure erythroid leukemia**	**BM**	**2.3**	**46,XX[20]**
**6**	**F**	**55**	**AML with inv(16)**	**PB**	**59**	**50,XX, add(7), +8, +14, der(16)t(11;16), inv(16)(p13.1q22), +20, +21 / 50,XX,add(7),+8,+14,add(16),+?20, +21[17]/46,XX[3]**
**7**	**F**	**80**	**AML without maturation**	**PB**	**35**	**46,XX,del(1)(p36.1),-7,add(9)(q12),-17,+2mar[13]/46,XX,add(9)(q22)[3]/46,XX[9]**
**8**	**M**	**26**	**CML BP**	**BM**	**30**	**45,X,-Y, t(9;22)(q34;q11.2)[20]**
**9**	**F**	**61**	**CML CP**	**BM**	**3**	**46,XX,t(9;22)(q34;q11.2)[20]**
**10**	**M**	**56**	**CML CP**	**BM**	**2**	**46,XY,t(9;22)(q34:q11.2)[20]**
**11**	**F**	**53**	**CML CP**	**BM**	**1**	**46,XX,t(9:22)(q34:q11.2)[20]**
**12**	**M**	**60**	**CML CP**	**PB**	**8**	**46,XY,t(9;22)(q34;q11.2)[20]**

AML, acute myeloid leukemia; CML, chronic myeloid leukemia; BP, blast phase; CP, chronic phase.

### Ethics statement

All subjects provided informed written consent before the commencement of the study. The study protocol, patient consent form, and accompanying information were approved by the Ulsan University Hospital Ethics Committee and Institutional Review Board (UUH-IRB-11-18).

### Isolation and culturing of patient cells

Patient cells were isolated by the density gradient method, as previously described [12]. Briefly, peripheral blood mononuclear cells (PBMCs) and bone marrow cells (BMCs) were isolated by the density gradient centrifugation at 400×g using Lymphoprep (Axis-Shield, Oslo, Norway), washed with PBS, and then cultured in the RPMI-1640 medium in 24-well culture plates with 10% FBS and 1% penicillin-streptomycin in a 5% CO_2_ humidified atmosphere at 37°C. The cells were then subjected to the following experiments described below.

### Cell viability assay

Cells were seeded in 96-well plates at a density of 2×10^4^ cells/ml with 100 μL of medium per well and then incubated with various concentrations of radotinib (0, 1, 10, and 100 μM) for 72 h at 37°C. The CellTiter 96 solution (20 μL) was added directly to each well and plates were incubated for 4 h in a humidified 5% CO_2_ atmosphere at 37°C. Absorbance was measured with a PowerWave XS2 Microplate Spectrophotometer (BioTek, Winooski, VT) at 490 nm and the results were expressed as percentage changes from the basal condition using four to five culture wells for each experimental treatment. In some experiments, HL60 cells were cultured with 100 nM ATRA and 1 μM dasatinib for 4 days, and 10 μM radotinib was added to each group according to the planned schedule.

### Detection of CD11b^+^Annexin V^+^ cells

Cells were incubated with different concentrations of radotinib (0, 1, 10 and 100 μM for BMCs; 0, 1, 5 and 10 μM for AML cell lines) for 72 h at 37°C, then harvested and washed twice with the FACS buffer (PBS containing 0.3% BSA and 0.1% NaN_3_). First, cells were stained with an anti-human CD11b-PE on ice for 30 min. After incubation, they were washed twice with the FACS buffer. Second, cells were incubated with Annexin V-FITC from the Apoptosis Detection Kit I (BD Bioscience, San Diego, CA) on ice for 30 min. Cells were then washed twice with the FACS buffer and analyzed using a FACSCalibur flow cytometer and CellQuest Pro software. Results were expressed as fold-changes of control values for each experimental condition. We also analyzed stained cells with the FlowSight and IDEAS software.

### Intracellular staining of cleaved caspase-3

Intracellular staining of cleaved caspase-3 in Kasumi-1 cells and BMCs from AML patients was carried out as previously described [12]. Briefly, CD11b^+^Annexin V^+^ cells were stained using the method detailed above. Next, they were fixed with 4% paraformaldehyde in PBS and 0.1% Triton X-100 was added for permeabilization. The cells were stained with an anti-cleaved caspase-3 monoclonal antibody (mAb) or an isotype control mAb at 4°C for 30 min. The samples were then analyzed with a FACSCalibur flow cytometer and CellQuest Pro software. Results were expressed as fold-changes of control values for each experimental condition. Stained cells were also analyzed with the FlowSight and IDEAS software.

### Immunoprecipitation and immunoblot analysis

NB4 and HL60 cells (1 × 10^7^ cells/ml) were incubated with 0, 5, or 10 μM radotinib or dasatinib for 48 h and lysed in 500 μL of the RIPA buffer for 30 min at 4°C. The extracts were pre-cleared with protein G-Sepharose and immunoprecipitated with 20 μg of anti-LYN. Protein G-Sepharose (50 μg) was added to cell extracts, which were then washed by centrifugation. Protein content was determined by the standard Bradford method. Equal quantities of solubilized proteins were resolved on 10% SDS-PAGE. Separated proteins were transferred to a nitrocellulose membrane and immunoblotted with an anti-phospho SFK mAb (Y416). The blots were then incubated with an anti-rabbit IgG-HRP and proteins were developed using the Immun-star WesternC kit. In some experiments, nitrocellulose membranes were stripped, reprobed with anti-ERK, c-Raf, or LYN mAb, and then incubated with respective secondary antibodies. Finally, the membranes were detected by the Immun-star WesternC kit.

### Microarray analysis

Kasumi-1 cells were grown in the presence or absence of 5 μM radotinib for 48 h and analyzed using a 44K Oligo Microarray (Agilent Technologies, Inc., Palo Alto, CA). Total RNA was isolated from cell samples using conventional TRIzol reagent and its integrity was measured using the Agilent’s Bioanalyzer 2100 RNA Nano kit. Synthesis of target cRNA probes and hybridization were performed using the Agilent Low RNA Input Linear Amplification kit PLUS according to the manufacturer’s instructions. Briefly, 1 μg of sample total RNA and T7 promoter primer mix were mixed and incubated at 65°C for 10 min. cDNA master mix (5× first strand buffer, 0.1 M DTT, 10 mM dNTP mix, RNase-Out, and MMLV-RT) was prepared and added to the mixture of RNA and primer. The samples were incubated at 40°C for 2 h for reverse-transcription and double-strand cDNA (dsDNA) synthesis and terminated by incubating at 65°C for 15 min. The transcription master mix was prepared according to the manufacturer’s protocol (4× transcription buffer, 0.1 M DTT, NTP mix, 50% PEG, RNase-Out, inorganic pyrophosphatase, T7-RNA polymerase, and Cyanine 3/5-CTP), added to the dsDNA reaction mixture, and incubated at 40°C for 2 h for the transcription of dsDNA. During transcription and amplification, cRNA samples were labeled with Cy3-CTP and Cy5-CTP, respectively. Amplified and labeled cRNA was purified and quantified using an ND-1000 spectrophotometer (NanoDrop Technologies, Inc., Wilmington, DE) to check labeling efficiency. The fragmentation of cRNA was performed by adding 10× blocking agent and 25× fragmentation buffer and incubation at 60°C for 30 min. Fragmented cRNA was resuspended with 2× hybridization buffer and directly pipetted onto microarray placed in the Hybridization Chamber (Agilent Technologies). The hybridization chamber was incubated at 42°C for 16 hours and mildly agitated to permit competitive hybridization reactions between labeled targets and probes on microarray. To eliminate non-specific binding, hybridized microarrays were washed with the Agilent’s Gene Expression Wash Buffer Kit (Agilent Technologies). Finally, microarrays were spin-dried and stored in the dark until scanning.

### Microarray Data Analysis

Scanned images were analyzed by the Feature Extraction program (Agilent Technologies). The average fluorescence intensity for each spot was calculated and local background was subtracted. All data manipulation, selection of fold-changed genes, and statistical Student’s *t*-test calculations were performed using GeneSpring 7.3.1 (Agilent Technologies). All numerical data were normalized by the intensity-dependent normalization (LOWESS), where the ratio was reduced to the residual of the Lowess fit of the intensity vs. ratio curve using GeneSpring 7.3.1 software. Genes with more than twofold change in the expression level were selected and considered as significant. For the expression pattern analysis, hierarchical clustering analysis was also performed using GeneSpring 7.3.1 software. Functional enrichment/grouping analyses were done using Gene Ontology (GO) functional classification system (www.geneontology.org) or DAVID (http://david.abcc.ncifcrf.gov/)

### Statistics

Data are expressed as the mean ± standard error of mean (SEM) of at least three independent experiments. All values were evaluated by one-way ANOVA followed by the Tukey’s range test using the GraphPad Prism 6.0 software. Differences were considered significant if *P* < 0.05.

## Results

### HPLC analysis and the structure of radotinib

**Fig 1:**
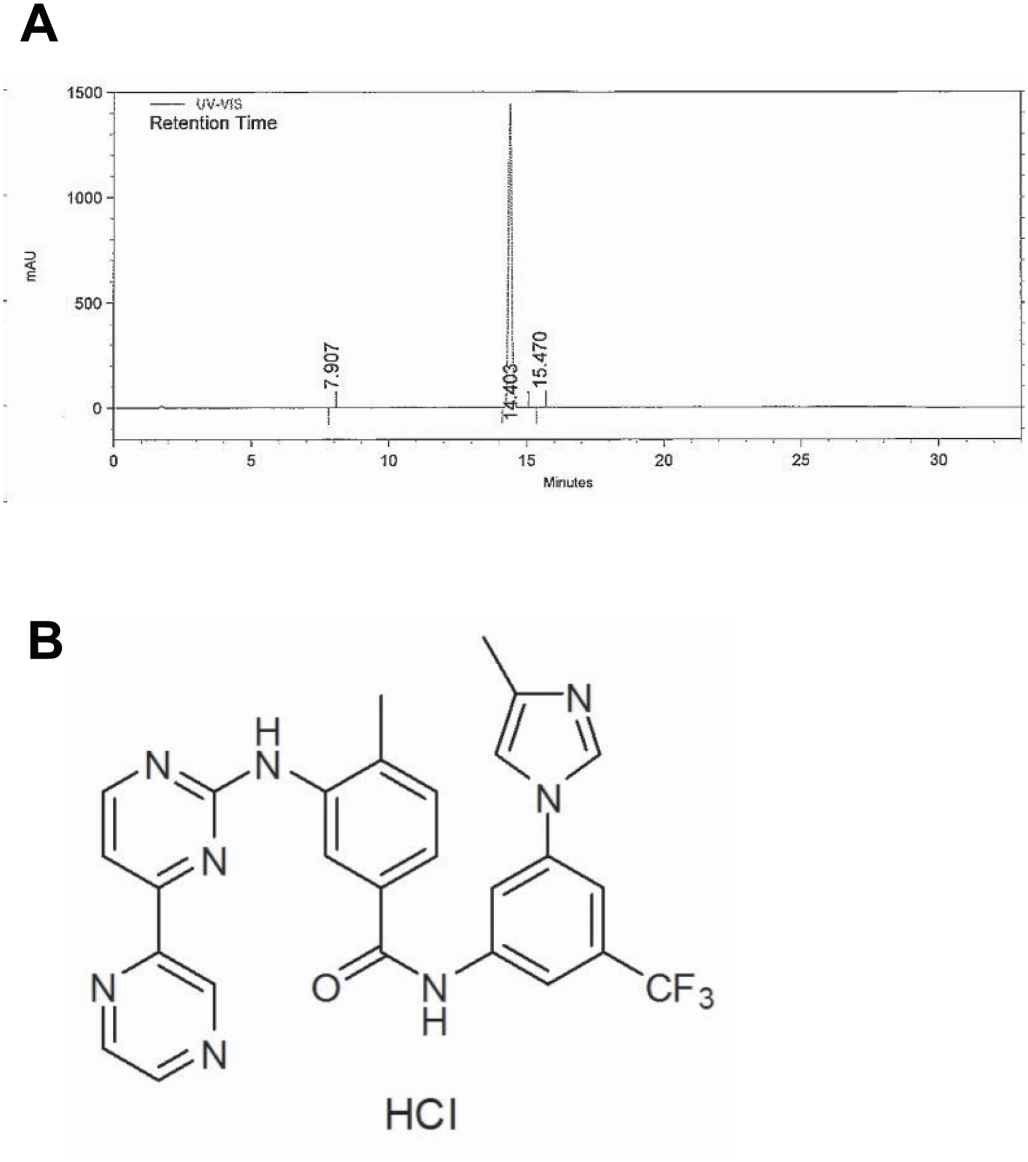
HPLC analysis and the structure of radotinib. (A) Radotinib was analyzed by HPLC as described in the Materials and Methods section. (B) The chemical structure of radotinib.

In accordance with the Certificate of Analysis, radotinib appeared as pale yellow crystalline powder. It was soluble in DMSO and partly soluble in methanol and ethanol. The purity of radotinib was ≥99% based on the HPLC analysis (Fig 1A). The chemical structure of radotinib is shown in Fig 1B.

### Radotinib induces AML cell death

Although radotinib was developed as a drug for the treatment of CML, it significantly decreased the viability of BMCs from AML patients in a dose-dependent manner after 48 h incubation, as shown in Table 2. In contrast, we failed to detect a decreased viability of BMCs from CML patients in the same conditions as above. Average values of cell viability at 100 μM radotinib comprised 62.6 ± 3.6% and 98.2 ± 5.7% for BMCs of AML and CML patients, respectively. The response was more prominent in BMCs than PBMCs of AML patients.

**Table 2:** Effects of Radotinib on the cell viability in patients with AML and CML.

UPN	Disease	Cell source	0 μM	1 μM	10 μM	100 μM
**1**	**AML**	**BM**	**100 ± 0.0**	**69 ± 1.2** ***	**58± 1.5** ***	**57 ± 2.3** ***
**2**	**AML**	**BM**	**100 ± 0.0**	**75 ± 1.0** ***	**73 ± 0.5** ***	**68 ± 0.9** ***
**3**	**AML**	**BM**	**100 ± 0.0**	**85 ± 2.5** ***	**78 ± 2.5** ***	**65 ± 2.7** ***
**4**	**AML**	**BM**	**100 ± 0.0**	**103 ± 6.5**	**64 ± 6.2** ***	**45 ± 3.5** ***
**5**	**AML**	**BM**	**100 ± 0.0**	**95 ± 0.9** **	**90 ± 0.9** ***	**78 ± 2.1** ***
**6**	**AML**	**PB**	**100 ± 0.0**	**91 ± 3.9**	**86 ± 6**	**87 ± 3** *
**7**	**AML**	**PB**	**100 ± 0.0**	**94 ± 0.6** **	**90 ± 0.8** **	**69 ± 1.8** ***
**8**	**CML**	**BM**	**100 ± 0.0**	**98 ± 7.8**	**88± 3.7**	**87 ± 6.3**
**9**	**CML**	**BM**	**100 ± 0.0**	**91 ± 5.5**	**95 ± 9.2**	**77 ± 2.9** **
**10**	**CML**	**BM**	**100 ± 0.0**	**96 ± 0.6**	**100 ± 1**	**112 ± 2**
**11**	**CML**	**BM**	**100 ± 0.0**	**99 ± 0.8**	**102 ± 0.4**	**113 ± 1**
**12**	**CML**	**PB**	**100 ± 0.0**	**101 ± 3.2**	**98 ± 1.4**	**91 ± 5**

These data represent the means ± SEM. Significantly different from control, 0 μM (*);

*: *P* < 0.05;

**: *P* < 0.01;

***: *P* < 0.001.

We also observed cytotoxic action of radotinib on four AML cell lines characterized by different genetic rearrangements as summarized in Table 3. NB4 cells belong to the M3 subtype according to the French-American-British (FAB) classification of AML and thus express the PML-RARA fusion protein [2]. Both Kasumi-1 and HL60 cells belong to the FAB M2 subtype, but they have different cytogenetic phenotypes, as only Kasumi-1 cells express the AML1-ETO fusion protein [2,15]. THP-1 cells belong to the FAB M5 subtype and exhibit the t(9;11)(p22;q23) translocation and MLL-AF9 fused oncogene expression [16]. Kasumi-1 cells exhibiting the t(8;21)(q22;q22) translocation were most sensitive of the four tested AML cell lines to low concentrations of radotinib. At the same time, NB4 cells expressing the t(15;17)(q22;q12) translocation were most sensitive to the high (100 μM) concentration of radotinib. The lowest cytotoxicity of radotinib was observed in HL60 cells, as shown in Table 3. Therefore, these results indicate that radotinib is able to induce AML cell death and the magnitude of its cytotoxic effect depends on the AML cell type.

**Table 3:** Effects of Radotinib on the cell viability in AML cell lines.

Cell lines	0 μM	1 μM	10 μM	100 μM	Karyotype
**NB4**	**100 ± 0.0**	**69 ± 1.8** ***	**61 ± 2.2** ***	**18 ± 2.3** ***	**t(15;17)(q22;q12)**
**HL60**	**100 ± 0.0**	**93 ± 8.6**	**84 ± 7.9** *	**52 ± 1.8** ***	**Hypotetraploid**
**KASUMI-1**	**100 ± 0.0**	**45 ± 1.3** ***	**45 ± 1.5** ***	**39 ± 5** ***	**t(8;21)(q22;q22)**
**THP-1**	**100 ± 0.0**	**98 ± 5**	**68 ± 2.2** ***	**46 ± 3.6** ***	**t(9;11)(p22;q23)**

These data represent the means ± SEM. Significantly different from control, 0 μM (*);

***: *P* < 0.001;

*: *P* < 0.05.

### Radotinib increases differentiation capacity of AML cells

**Fig 2:**
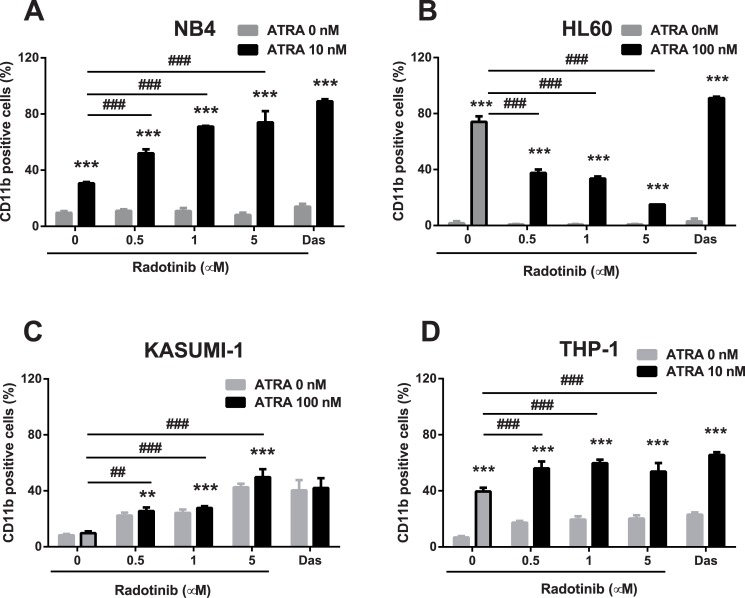
Radotinib induces CD11b expression in AML cells. Cells were incubated with various concentrations of radotinib and/or ATRA for 72 h, harvested and immunostained with an anti-human CD11b mAb. The expression of CD11b was measured by flow cytometry in NB4 (A), HL60 (B), Kasumi-1 (C), and THP-1 cells (D). Data are presented as the mean ± SEM. Statistically significant differences from the DMSO-treated control (*) or ATRA treatment (#) are denoted as follows. **, ##: *P* < 0.01; ***, ###: *P* < 0.001. Das, dasatinib; ATRA, all-trans retinoic acid.

We examined effects of radotinib on the expression of the cell surface differentiation marker CD11b^+^. The cells were treated with different concentrations of radotinib for 72 h and the expression of the differentiation marker was analyzed by flow cytometry. As shown in Fig 2A–2D, radotinib-induced CD11b^+^ expression was increased in NB4 and THP-1 cells when combined with ATRA treatment. In Kasumi-1 cells, incubation with radotinib alone induced CD11b^+^ expression and ATRA had no additive effect on the expression of this marker. Moreover, ATRA-induced CD11b^+^ expression was actually decreased by radotinib in HL60 cells (Fig 2B and 2C). Also, we studied the action of dasatinib on CD11b^+^ expression in all four cell lines and compared it to effects of radotinib. Patterns of regulation of CD11b^+^ expression by these two drugs were very similar in NB4, Kasumi-1, and THP-1 AML cells, however their modulatory effects were opposite in HL60 cells: CD11b^+^ expression was decreased by radotinib in ATRA-treated HL60 cells, whereas it was increased by dasatinib (Fig 2A–2D).

### Radotinib-induced differentiation of AML cells is related to downregulation of LYN activity

**Fig 3:**
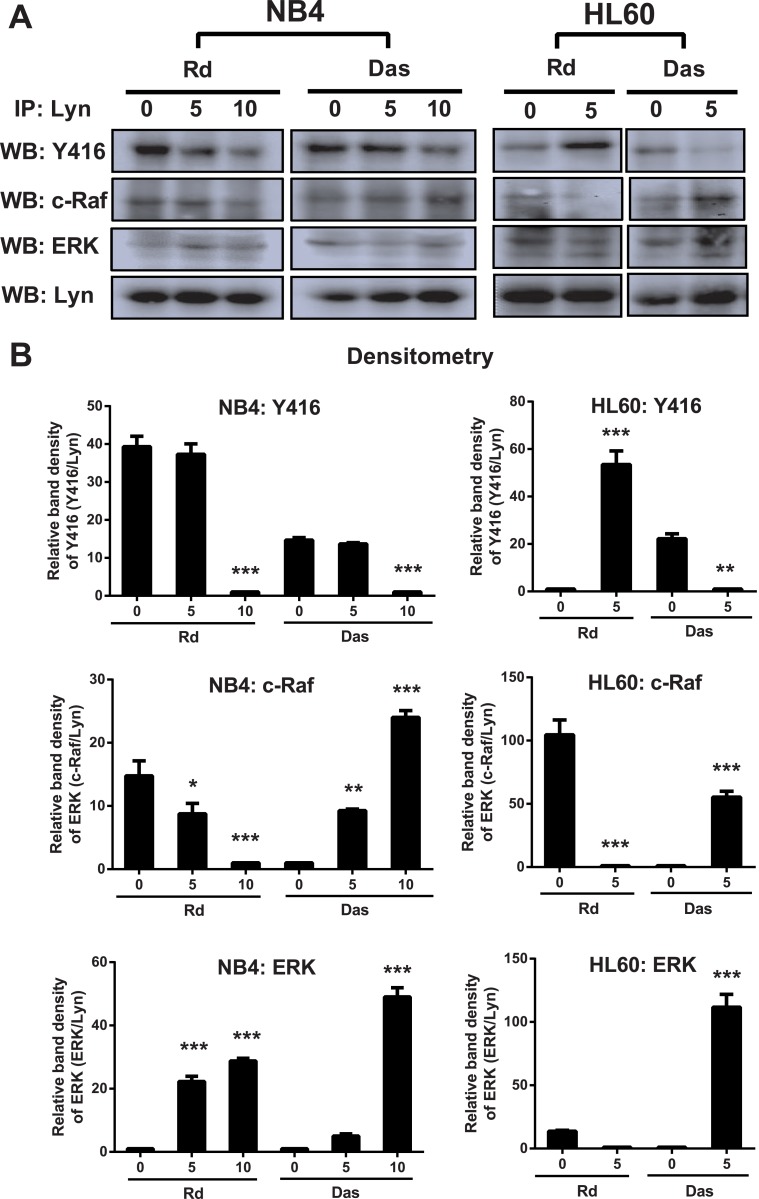
Differentiation capacity of CD11b^+^ cells is enhanced by the inhibition of LYN activity. (A) Radotinib decreased LYN activity in NB4 cells but increased it in HL60 cells. Cells were stimulated with 0, 1, and 5 μM radotinib or dasatinib for 48 h. After immunoprecipitation of LYN, immunoprecipitate pellets were subjected to immunoblotting with antibodies against phosphor-SFK (Y416), c-Raf, ERK, and LYN in NB4 and HL60 cells. (B) The relative band density is shown. Data are presented as the mean ± SEM. Statistically significant differences from the DMSO-treated control are given as follows. **: *P* < 0.01; ***: *P* < 0.001. Rd, radotinib; Das, dasatinib.

We sought to determine the mechanism of differential regulation of CD11b^+^ expression in HL60 cells by radotinib and dasatinib. Thus, we examined changes in the activity of LYN in NB4 and HL60 cells following radotinib stimulation. After immunoprecipitation of LYN, immunoprecipitate pellets were subjected to immunoblotting with an anti-phospho-SRC family kinase antibody. Western blotting results in NB4 cells showed that Y416 active site phosphorylation levels were dose-dependently decreased by both radotinib and dasatinib (Fig 3A, left panel). Thus, decreased LYN activity by radotinib in NB4 cells enhanced CD11b^+^ expression and contributed to myeloid differentiation. However, the expression of phosphorylated Y416 in HL60 cells was increased by radotinib but decreased by dasatinib (Fig 3A, right panel). Moreover, we found that LYN was likely to be an important target of radotinib action, as it was involved in signaling cascades by binding to c-Raf and extracellular signal-regulated kinase (ERK). These findings agreed with contrasting changes in the CD11b^+^ expression pattern in NB4 and HL60 induced by radotinib (Fig 2A and 2B). Therefore, these results indicate that radotinib-induced AML cell differentiation (or CD11b^+^ expression) is closely related to the downregulation of Lyn activity (Fig 3A).

### Radotinib induces CD11b^+^Annexin V^+^ cells in AML

**Fig 4:**
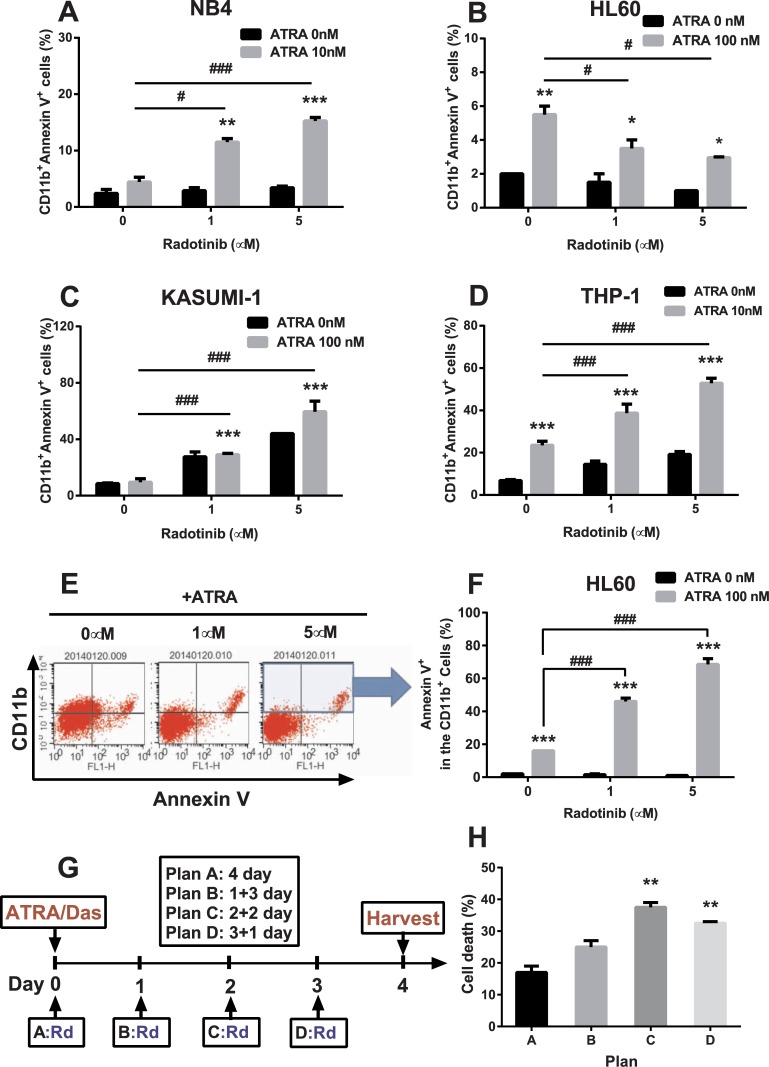
Radotinib induces CD11b^+^Annexin V^+^ cells in AML cell lines. Cells were incubated with various concentrations of radotinib and/or ATRA for 72 h, harvested and immunostained with anti-human antibodies against CD11b and Annexin V, as described in the Methods. (A) NB4. (B) HL60. (C) Kasumi-1. (D) THP-1. (E) The CD11b^+^Annexin V^+^ cells in HL60. (F) Annexin V^+^ in the CD11b^+^ gated cells. (G) Schedule of differentiation induced cell death (plans A, B, C and D). (H) Cell death data by the respective plan. Data represent the mean ± SEM. Statistically significant differences from the DMSO-treated control (*) or ATRA treatment (#) are denoted as follows.*, #: *P* < 0.05; **, ##: *P* < 0.01; ***, ###: *P* < 0.001. Rd, radotinib; Das, dasatinib; ATRA, all-trans retinoic acid.

We have been searching for a possible link between cell differentiation and AML cell death for a long time. In our previous study, we revealed that differentiating CD11b^+^ cells in AML tend to become apoptotic [12]. Therefore, the ability to regulate the differentiation capacity is a very useful instrument to control AML cell death. Thus, CD11b^+^Annexin V^+^ cells can be a marker for measuring AML blasts death by means of increased differentiation capacity. We considered that this phenomenon could stimulate differentiation-dependent cell death in AML. The cells were stimulated by radotinib with or without ATRA for 72 h and CD11b^+^Annexin V^+^ cells were monitored. We found that numbers of CD11b^+^Annexin V^+^ cells in radotinib/ATRA-treated cultures were 6.4-fold, 1.5-fold, 7-fold, and 7.7-fold higher than those of the control group at 72 h in NB4 (Fig 4A), HL60 (Fig 4B), Kasumi-1 (Fig 4C), and THP-1 cells (Fig 4D), respectively.

**Fig 5:**
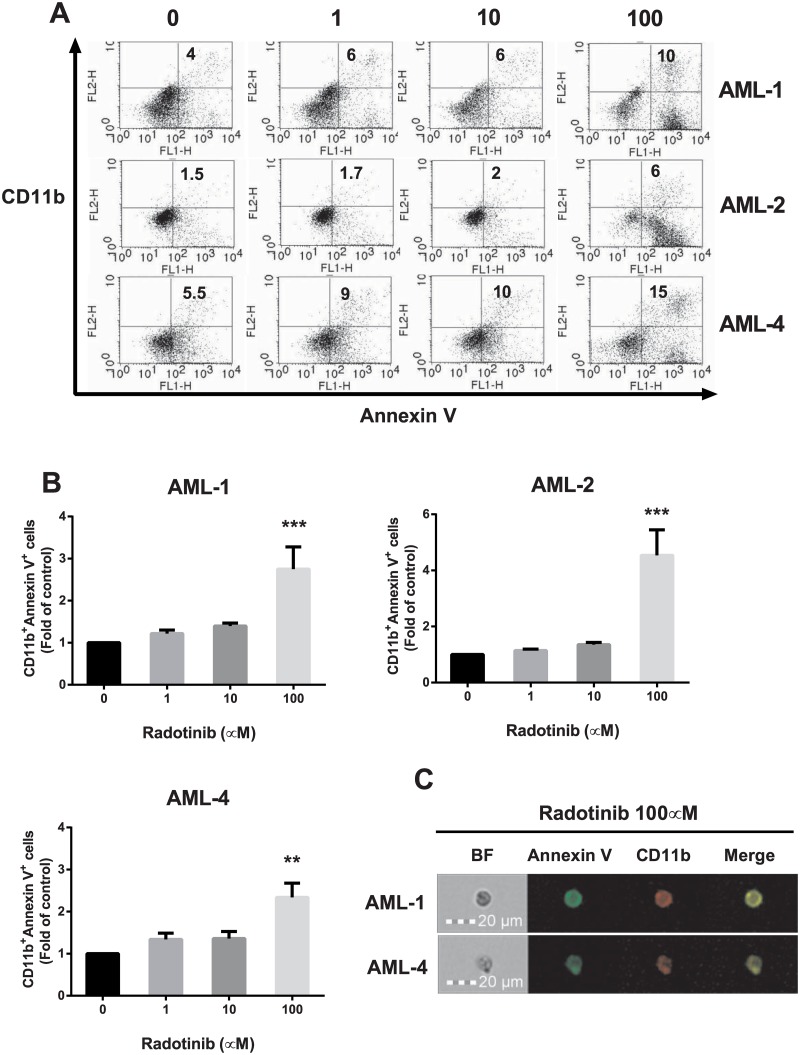
Radotinib induces CD11b^+^Annexin V^+^ cells in BMCs from patients with AML. Cells were incubated with various concentrations of radotinib for 72 h, harvested and immunostained with anti-human antibodies against CD11b and Annexin V. (A) The CD11b^+^Annexin V^+^ cells in AML-1, AML-2 and AML-4. (B) Data show the fold-change of the number of CD11b^+^Annexin V^+^ cells compared to control conditions in AML-1, AML-2, and AML-4. (C) The expression of Annexin V and CD11b in cells treated with 100 μM radotinib was monitored by FlowSight analysis (Patients: AML-1 and AML-4). Data represent the mean ± SEM. Statistically significant differences from the DMSO-treated control (*) are denoted as follows. **: *P* < 0.01; ***: *P* < 0.001. BF, bright field.

Next, we examined numbers of CD11b^+^Annexin V^+^ cells in the total population of BMCs from AML patients by flow cytometry as shown in Fig 5A. The results summarized in Fig 5B had the same pattern as in Kasumi-1 cells. Average number of CD11b^+^Annexin V^+^ cells among BMCs of AML patients (AML-1, AML-2, and AML-4) following 100 μM radotinib treatment was 3.2-fold higher than in the control group at 72 h. Also, we confirmed the expression against CD11b^+^Annexin V^+^ cells in BMCs of AML patients (AML-1 and AML-4) after 100 μM radotinib stimulation as shown in Fig 5C. These results indicate that radotinib induces CD11b^+^Annexin V^+^ cells by differentiation induced cell death in AML.

### Differentiation is associated with AML cell death by radotinib

We analyzed the number of Annexin V^+^ cells among differentiated CD11b^+^ cells and observed that this cell population was dose-dependently increased by radotinib in HL60 cells in presence of ATRA (Fig 4E and 4F). We then realized that the levels of cell death were correlated with the expression of the differentiation marker CD11b^+^ according to the previous results (Table 2, Table 3, and Figs 2–5). We hypothesized that pre-differentiated cells would be more susceptible to radotinib-induced AML cell death and planned a series of experiments to confirm this hypothesis. HL60 cells were incubated with 100 nM ATRA and 1 μM dasatinib for 4 days, and then 10 μM radotinib was added daily according to a planned schedule as shown in Fig 4G. As we expected, there was a higher cell death percentage after 2 or 3 days of differentiation latency upon treatment with ATRA/dasatinib (Fig 4H). Therefore, radotinib not only induces CD11b^+^Annexin V^+^ cells but also enhances AML cell death by potentiating differentiation. Moreover, differentiation may help immature cells to develop into more mature (CD11b^+^ macrophages-like) cells and consequently to become more susceptible to deadly stimuli. These results illustrate differentiation-dependent cell death in AML cells (Figs 4 and 5).

### Radotinib induces mitochondrial depolarization and caspase-3 dependent apoptosis of CD11b^+^ AML cells

**Fig 6:**
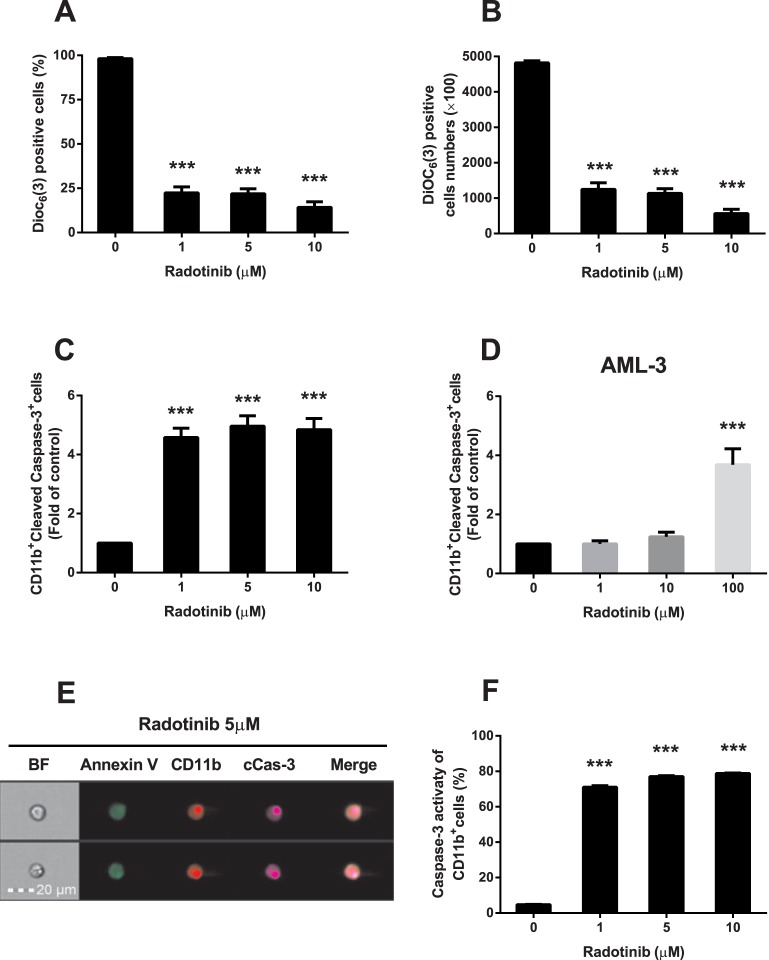
Radotinib induces caspase-3 dependent apoptosis of CD11b^+^ cells differentiated from AML cells. Kasumi-1 cells were incubated with 0, 1, 5, and 10 μM of radotinib, and BMCs from a patient with AML were stimulated with 0, 1, 10 and 100 μM of radotinib for 72 h. Then the cells were harvested and used for measurements of the mitochondrial membrane potential using Dioc_6_(3) dye, intracellular staining for cleaved caspase-3, or studies of the caspase-3 activity in CD11b^+^ cells, as described in Materials and Methods. (A) The percentage of Dioc_6_(3)^+^ cells in the total population of CD11b^+^ cells. (B) The number of Dioc_6_(3)^+^ cells among CD11b^+^ cells. (C) CD11b^+^cleaved caspase-3^+^ cells among Kasumi-1 cells. (D) CD11b^+^ cleaved caspase-3^+^ cells among BMCs isolated from an AML-3 patient. (E) The expression of Annexin V, CD11b, and cleaved caspase-3 induced by 5 μM radotinib in Kasumi-1 cells was monitored by FlowSight analysis. (F) Caspase-3 activity in CD11b^+^ Kasumi-1 cells. Data represent the mean ± SEM. Statistically significant differences from the DMSO-treated control (*) are denoted as follows. ***: *P* < 0.001. BF, bright field; cCas-3, cleaved caspase-3.

We examined effects of radotinib on the mitochondrial membrane potential (*ΔΨ*) and caspase-3 activity. CD11b^+^ cells were collected and the mitochondrial membrane potential was measured by flow cytometry using Dioc_6_(3) dye. Kasumi-1 cells were the most sensitive to radotinib treatment among the four AML cell lines tested (Table 3). Therefore, we investigated the activation of the apoptotic signaling pathway by radotinib in Kasumi-1 cells. As shown in Fig 6A, Dioc_6_(3)^+^ CD11b^+^ cells were markedly suppressed by radotinib stimulation. Notably, the absolute number of Dioc_6_(3)^+^ cells was similar under radotinib stimulation (Fig 6B). In contrast, the number of CD11b^+^/cleaved caspase-3^+^ cells was increased dose-dependently by radotinib in total populations of Kasumi-1 cells (Fig 6C) and BMCs (AML-3, Fig 6D). We carried out triple staining of Kasumi-1 cells by mAbs against Annexin V, CD11b, and cleaved caspase-3 following 72 h incubation of cells with radotinib as demonstrated by Flowsight images shown in Fig 6E. Next, we uncovered upregulation of caspase-3 activity by radotinib in CD11b^+^ cells (Fig 6F). We concluded that radotinib treatment results in a loss of the mitochondrial membrane potential and induction of caspase-3 dependent apoptosis of CD11b^+^ cells (Fig 6).

### Radotinib regulates many signaling pathways

**Fig 7:**
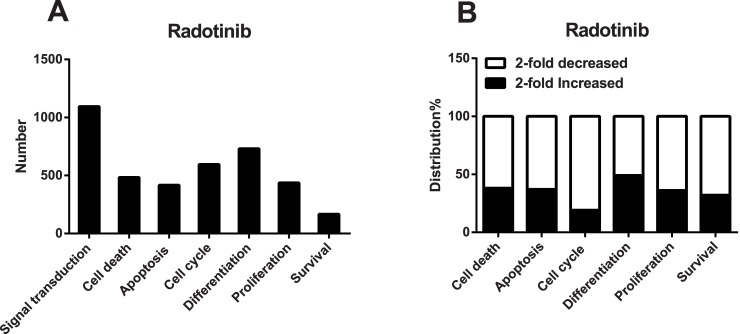
Radotinib regulates several signaling pathways. Kasumi-1 cells were incubated with 5 μM radotinib for 48 h. Cells were harvested, total RNA was isolated and subjected to microarray analysis, as described in the Materials and Methods. (A) The number of genes in categorized pathways affected by radotinib. (B) The percentage of distribution of radotinib-affected genes in each pathway.

A lot of genes were affected by 5 μM radotinib treatment of Kasumi-1 cells as shown in Fig 7A. We divided functions of proteins encoded by the affected genes into several categories including signal transduction, cell death, apoptosis, cell cycle, differentiation, proliferation, and survival. Thereafter, we assessed the number of pathways affected by radotinib. We noted that a significant number of genes encoding proteins involved in the G_1_ phase, e.g., cyclin-dependent kinases (*CDK2* and *CDK4* genes), were downregulated by radotinib according to microarray analysis data. On the contrary, apoptotic signaling pathway-related genes (*MDM4*, *BBC3*, *BCL2L11*, and *TP53*) were often upregulated by radotinib (Fig 7B). Many differentiation-related genes were affected by radotinib as well. Our results provide important clues for understanding mechanisms of action of radotinib.

## Discussion

Originally, imatinib (STI571, Gleevec), nilotinib, and dasatinib, which belong to TKIs, were developed for treatment of CML [9,17,18]. There have been several reports that these drugs were also very effective in AML and acute lymphoblastic leukemia (ALL). In short, imatinib affected degradation of RARα and PML-RARα in APL cells, a subtype of AML. Nilotinib was found to induce cytotoxic action in both Philadelphia-positive and Philadelphia-negative ALL cells [19]. Dasatinib proved to be effective in promoting differentiation and cell death of AML cells [12,20]. These data suggested that radotinib might also have possible off-target effects on AML cells. We hypothesized that radotinib could either induce differentiation or cause death of AML cells. Therefore, we set out to examine the anti-leukemic effect of radotinib and identify its mechanism of action in AML cells. Interestingly, radotinib induced AML cell death in BMCs and PBMCs from patients with AML and in AML cell lines. The relative proportion of CD11b^+^ cells was increased by radotinib in all types of AML cells and cell lines with the exception of HL60 cells (Figs 2–5).

It has been known that differentiation of AML cells is associated with Src family kinases (SFK) activity [21]. SFKs are important signaling molecules mediating responses to various signals including proliferation, differentiation, apoptosis, migration, and metabolism [22]. LYN, one of the SFK members, is an important regulator of autoimmune diseases such as asthma and psoriasis. In addition, LYN is important for maintaining the leukemic phenotype of various leukemic disorders including AML, CML, and B-cell lymphocytic leukemia [22,23]. Moreover, LYN is expressed in some solid tumors and serves as a potential therapeutic target for colon, prostate, glioblastoma, and more aggressive subtypes of breast cancer [24–27]. Generally, LYN is considered to play a role in the signal initiation and it also serves as a signaling intermediate [28]. According to Kropt et al., LYN is an essential molecule in the ATRA/dasatinib-induced AML differentiation [2]. Treatment with dasatinib, promotes tighter association of LYN with c-Raf and ERK [11] and we confirmed this earlier finding in experiments illustrated in Fig 3. Inhibition of SFK activity also has been effective in slowing leukemic cell growth and enhancing differentiation of AML cells [11,21,29]. Differentiation signals in AML cells were associated with the activity of LYN and mitogen-activated protein kinases (MAPK) [11]. Therefore, we examined the activity of LYN in NB4 and HL60 cells because the expression of the differentiation marker CD11b was increased by radotinib in ATRA-treated NB4 cells, while it was decreased by radotinib in ATRA-treated HL60 cells. Augmentation of differentiation signals of AML cells by radotinib, as evidenced by the increasing proportion of CD11b^+^ cells, was associated with the inhibition of LYN kinase activity in NB4 cells. On the contrary, downregulation of differentiation signals of AML cells by radotinib, i.e., decrease in CD11b^+^ cells, was associated with the activation of LYN kinase activity in HL60 cells. Collectively, these results suggest that radotinib induces CD11b expression in AML cells and possibly increases differentiation capacity by inhibiting the kinase activity of LYN (Figs 2 and 3).

Moreover, radotinib induced differentiation-dependent cell death in AML. Radotinib increased numbers of CD11b^+^Annexin V^+^ cells in AML cell lines and BMCs from patients with AML (Figs 4 and 5). Even though ATRA-induced CD11b^+^ cells of HL60 decreased after drug treatment, CD11b^+^Annexin V^+^ cells were upregulated by radotinib in a dose-dependent manner (Fig 4E and 4F). We investigated differentiation induced cell death in AML cells as shown in Fig 4G. To assess effects of radotinib on this process, HL60 cells were first incubated with ATRA/dasatinib and then stimulated with radotinib at after various delay periods. Late starting points of radotinib administration were associated with larger extent of cell death compared to treatment at early starting points (Fig 4H). These observations suggested that pre-conditioning differentiation could facilitate the induction of cell death. Differentiation therapy may help develop immature blasts to matured cells (CD11b^+^ macrophages), which could have a normal life span, develop into chemosensitive cells [30] and, finally, become prone to cell death.

Next, we examined the signaling pathway of the differentiation induced cell death. Mitochondria are the major source of metabolic energy in cells. Most functions of mitochondria depend on the membrane potential generated and maintained by their inner membrane [31]. The mitochondrial membrane potential also serves as a mediator in the apoptotic pathway. We collected CD11b^+^ cells and measured their mitochondrial membrane potential and caspase-3 related signals following radotinib stimulation. Radotinib not only disrupted the mitochondrial membrane potential but also induced caspase-3 activation in Kasumi-1 cells and BMCs of patients with AML. Therefore, radotinib induces differentiation induced cell death via mitochondrial depolarization and caspase-3 dependent apoptosis, particularly in the population of CD11b^+^ AML cells (Fig 6).

According to our observations, radotinib and dasatinib exerted comparable effects on AML cells, except in the case of HL60 cells. For example, induction of CD11b^+^ cells, association of LYN with ERK, and stimulation of death signals (data not shown) were similar between the two treatments. We cannot easily explain why radotinib and dasatinib had opposing effects on the CD11b expression in HL60 cells. To explore this discrepancy further, we plan to carry out a comparative study of effects of radotinib and dasatinib on c-Kit positive and c-Kit negative cells.

G_1_ phase-related genes, such as *CDK2* and *CDK4*, were downregulated by radotinib, while genes encoding proteins involved in apoptotic signaling pathways were upregulated (Fig 7). We will address the role of cell cycle related proteins and radotinib effects on the cell cycle distribution in further studies. At this stage, we can conclude that our results provided important clues for understanding the mechanisms of radotinib effects and shed some light on differential sensitivity of AML cell lines to this drug.

Finally, we found that CD11b^+^ cells differentiated from all types of AML cells were susceptible to cell death. We have not established whether differentiated cells died spontaneously or via activation of an apoptotic pathway directly activated by radotinib. In our opinion, both mechanisms may contribute to the AML cell death. Therefore, on the basis of our findings, radotinib may be proposed as a promising anti-cancer drug or a chemosensitizer for AML therapy.
